# Environmental enrichment normalizes hippocampal timing coding in a malformed hippocampus

**DOI:** 10.1371/journal.pone.0191488

**Published:** 2018-02-02

**Authors:** Amanda E. Hernan, J. Matthew Mahoney, Willie Curry, Greg Richard, Marcella M. Lucas, Andrew Massey, Gregory L. Holmes, Rod C. Scott

**Affiliations:** 1 Department of Neurological Sciences, University of Vermont College of Medicine, Burlington, Vermont, United States of America; 2 Department of Neurology, Geisel School of Medicine at Dartmouth, Lebanon, New Hampshire, United States of America; 3 University College London, Institute of Child Health, London, United Kingdom; Tokai University, JAPAN

## Abstract

Neurodevelopmental insults leading to malformations of cortical development (MCD) are a common cause of psychiatric disorders, learning impairments and epilepsy. In the methylazoxymethanol (MAM) model of MCDs, animals have impairments in spatial cognition that, remarkably, are improved by post-weaning environmental enrichment (EE). To establish how EE impacts network-level mechanisms of spatial cognition, hippocampal *in vivo* single unit recordings were performed in freely moving animals in an open arena. We took a generalized linear modeling approach to extract fine spike timing (FST) characteristics and related these to place cell fidelity used as a surrogate of spatial cognition. We find that MAM disrupts FST and place-modulated rate coding in hippocampal CA1 and that EE improves many FST parameters towards normal. Moreover, FST parameters *predict* spatial coherence of neurons, suggesting that mechanisms determining altered FST are responsible for impaired cognition in MCDs. This suggests that FST parameters could represent a therapeutic target to improve cognition even in the context of a brain that develops with a structural abnormality.

## Introduction

**Fig 1:**
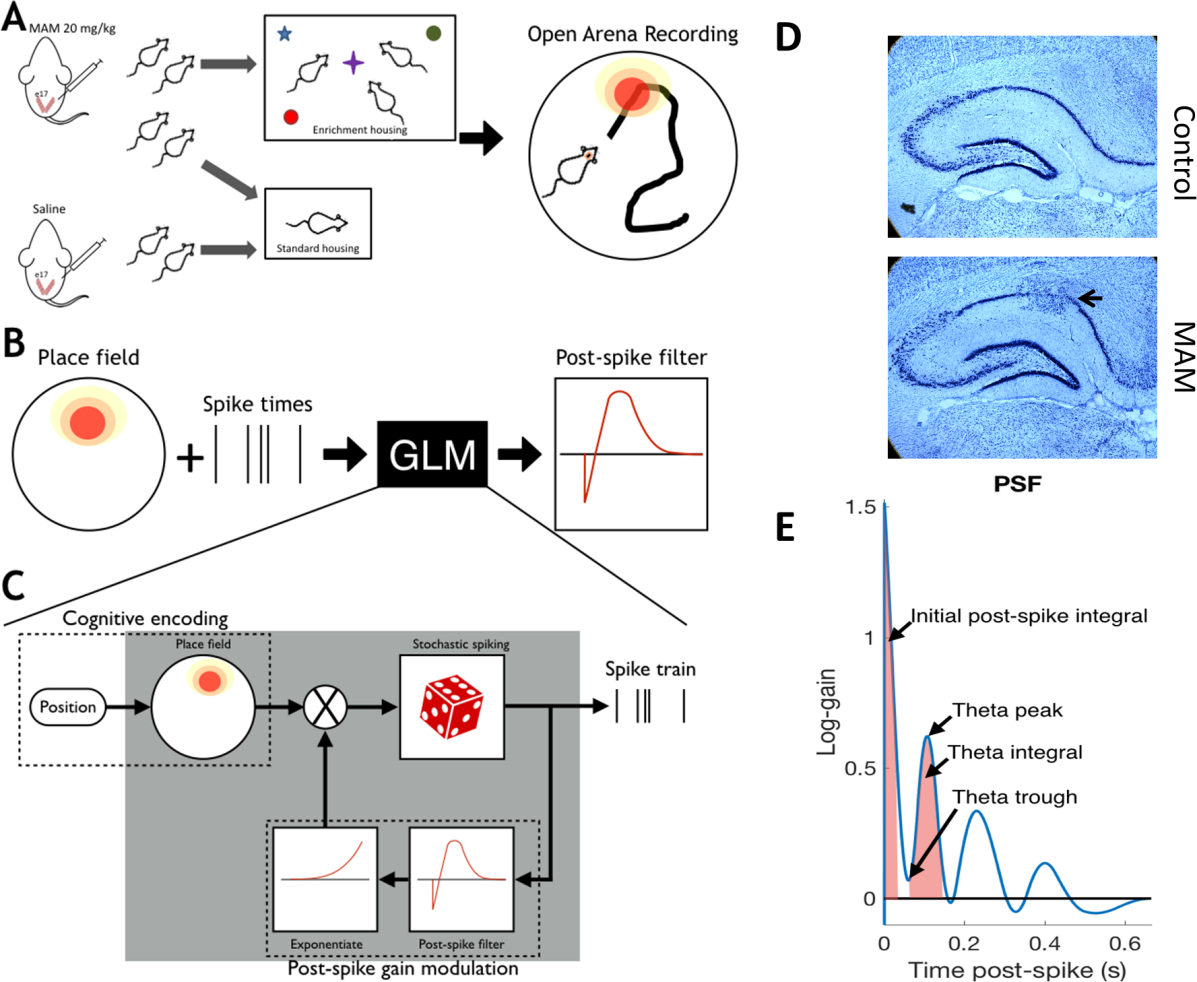
Environmental enrichment and analysis pipeline. (a) Pregnant dams were injected I.P. with methylazoxymethanol acetate (MAM) or saline (Control) at embryonic day 17. Post-weaning, control pups and one group of MAM pups were housed in standard conditions, while another group of MAM animals received environmental enriched (EE), including multiple novel objects to explore and other animals with which to interact. All animals were implanted as adults (>p60) with four tetrodes in CA1 and were recorded foraging in a familiar environment for food reward. Place fields were constructed by comparing spike times to the animal’s position acquired from video tracking. (b) Place fields of CA1 pyramidal cells and spike times were used in a generalized linear model (GLM) to model the fine-spike timing of cells, which is captured with a post-spike filter (PSF). Specifically, the PSF models the short timescale (< 650 ms) post-spike modulation of firing probability and captures the autocorrelation structure of cell firing. (c) As a stochastic model, the GLM considers the firing probability to be a function that varies in time. The cognitive component of the GLM uses the place field of the cell(top left). To capture the fast timescale structure, the GLM uses a PSF, which feeds back through an exponential nonlinearity to modulate the firing probability (bottom right). The PSF is defined by 11 parameters that are chosen to maximize the likelihood of observing the measured spike train. Thus, the PSF is learned through the GLM with input from the place field and the raw spike train. Note that the modulation by the PSF is multiplicative. Hence, the PSF corresponds to ‘gain modulation’. The PSF is parametrized by a set of basis functions. The fitted PSF is learned from the measured spike train (top right) through penalized maximum likelihood estimation. (d) Thionin stained coronal sections show stereotypical histology from control (top panel) and MAM (bottom panel) animals. Hippocampal dysplasias (arrow) can be seen after MAM treatment as well as some over-dispersion of pyramidal neurons in the cell layer of CA3. (e) Shows a representative PSF indicating parameters measured in the fine spike timing analysis. The GLM encodes the timing properties of neurons in a post-spike filter (PSF). The PSF account for the autocorrelation structure of firing, which cannot be accounted for with the place field alone. We analyze the fine spike timing using parameters derived from the PSF: initial post-spike integral and theta integral, which are areas under the PSF curve that account for up-regulation immediately after a spike and after one theta cycle, respectively, and theta peak and theta trough, which account for the peak up- and down-regulation of firing in theta.

Neurodevelopmental insults are common etiologies in many neurological diseases, including epilepsy, schizophrenia, cerebral palsy, and autism spectrum disorders. Malformations of cortical development (MCDs) are one result of an insult during neurodevelopment and are commonly identified in patients with significant cognitive impairments and seizures [1]. Cognitive impairments are a major driver of impaired quality of life and therefore strategies that maximize cognition would be expected to have a major positive impact on outcomes. In the context of epilepsy, the main therapeutic strategies have targeted seizures in order to try to achieve cognitive improvements. Unfortunately, this approach has had limited success, raising the issue of whether abnormal neural networks underlying cognitive impairments can be functionally modified to improve cognition independently of treating seizures. We have previously addressed this issue behaviorally in the methylazoxymethanol (MAM) model of MCDs in rats. *In utero* exposure of methylazoxymethanol acetate (MAM) produces offspring with neuropathology similar to patients with MCDs associated with early onset epilepsy (Fig 1). These animals display significant impairments in spatial learning and memory in both a water maze task as well as a spatial place avoidance task [2]. Remarkably, these impairments are dramatically improved following environmental enrichment [2], suggesting that brains with MCDs can be functionally altered to improve behavior. However, the nature of the underlying neural network changes responsible for this improvement is unknown. Extrapolating from environmental enrichment to a broader class of therapies targeting cognitive impairment in MCDs is impossible, given that there is no obvious target. Characterization of the system-level mechanisms of improvement following environmental enrichment could provide critical insight for development of novel therapies that improve cognition in patients with MCDs.

Information processing in the brain is a function of rhythmic dynamics, conceptualized here as system-level mechanisms of cognition. The hippocampus is a multi-layered series of integrated networks that underlie spatial cognition [3] and therefore modifications to the hippocampal neural system in MAM exposed animals with MCDs could explain both the spatial cognition impairments and the observed improvements following environmental enrichment. Pyramidal cell action potential firing within these networks is precisely modulated both in space and in time with respect to neuronal oscillations in the local field potential [4]. Modulation of firing rate (rate coding) with respect to spatial location is observed with hippocampal place cells. Modulation of action potential firing with respect to time (timing coding) is observed, e.g., in the phenomena of phase preference and phase precession [5]. The rate and timing modulations of pyramidal cell firing dynamics are sculpted by a wide variety of interneurons, including those targeting the axon initial segment and predominantly expressing parvalbumin. This precise modulation of pyramidal cell firing with respect to space and time is crucial for information processing, resulting in effective spatial learning and memory [6]. We hypothesize that MCDs are associated with abnormalities in rate and timing coding in hippocampal CA1 pyramidal cells, and that environmental enrichment, which is therapeutically effective in this model, will improve these functional abnormalities, even in the context of a malformed brain (see Fig 1).

Both rate and timing coding influence the probability of an action potential (*spike*) at any given time. Typical summaries of spike trains, such as autocorrelograms (ACGs), are complex mixtures of all effects that dynamically modulate firing. Therefore, to dissociate rate and timing effects from each other, we used a generalized linear modeling (GLM) approach that incorporates both a neuron’s spatial receptive field and its timing properties (Fig 1B and 1C). The GLM approach is a statistically principled way to extract structure from spike trains that explicitly models timing coding over-and-above spatial rate coding. The GLM captures timing features robustly without, for example, requiring a minimum firing rate for analysis. Using the GLM, we are able to extract timing features that can then be quantitatively related back to rate coding parameters without *ad hoc* summaries of spiking dynamics or conceptual circularity inherent in trying to examine temporal coding from the ACG alone.

We show for the first time that rate and timing coding in hippocampal CA1 pyramidal cells is disrupted in MAM animals and that this disruption is associated with a reduction in parvalbumin expressing interneurons. Environmental enrichment modifies rate and timing modulation disruptions and increases the number of parvalbumin expressing interneurons, suggesting that abnormal networks can be functionally improved. This supports the view that strategies that normalize pyramidal cell firing dynamics have substantial therapeutic promise for patients with MCDs.

## Results

In order to understand the neural networks underpinning poor spatial cognition and improved cognition after environmental enrichment (EE) in MAM animals, we used *in vivo* electrophysiological tetrode recording of pyramidal cells in CA1 of the hippocampus (Fig 1). We monitored three groups of animals during foraging in an open field: 1) Controls, 2) MAM animals (MAM), and 3) MAM animals exposed to post-weaning environmental enrichment (MAM-E). To determine the effects of MAM and EE on neural circuits, we studied 258 hippocampal CA1 pyramidal cells from 15 rats: 4 controls (59 cells), 7 MAM unenriched (101 cells), and 4 MAM-E (98 cells). Table 1 provides information regarding standard electrophysiological properties of all pyramidal neurons recorded in our cohort. As a whole population, the action potential half width (p = 0.307, Wald Chi-Square 2.365) and mean firing rate during the session (p = 0.12, Wald-Chi Square 4.297) was not different between the groups, indicating homologous populations of neurons were recorded from all groups of animals (Table 1). Instantaneous frequency was different between the groups (p = 0.001; Wald-Chi square 14.540, Cohen’s d 0.49 MAM vs MAM-E, 0.072 MAM vs controls), with neurons from MAM animals having a slightly lower instantaneous frequency overall than controls or MAM-E animals. Behaviorally, the spatial coverage (measured in bits) as the “fraction" of the arena covered by an animal during the session is not different between the groups (kruskal-wallis, p = 0.982), nor is average speed (in pixels per second) of each animal (kruskal- wallis p = 0.23).

**Table 1:** Description of the standard firing characteristics of all recorded populations of pyramidal neurons. The table breaks characteristics down by cell firing properties (bursty cells in normal-type font the top three rows, refractory cells italicized in the bottom three rows) and by group (Control shaded in blue, MAM in red, MAM-E in beige).

		Spatial Coherence		Spike Width		Mean Firing Rate		Instantaneous Firing Frequency	
Bursty	Control	0.2967 ±	0.023	0.3172 ±	0.017	0.4823 ±	0.109	25.2727 ±	4.693
	MAM	0.2213 ±	0.012	0.3503 ±	0.022	0.7745 ±	0.163	25.4459 ±	2.568
	MAM-E	0.323 ±	0.034	0.3587 ±	0.030	0.7626 ±	0.026	36.4773 ±	3.751
*Refractory*	*Control*	*0*.*1165 ±*	*0*.*034*	*0*.*3021 ±*	*0*.*023*	*1*.*0822 ±*	*0*.*304*	*10*.*5183 ±*	*1*.*744*
	*MAM*	*0*.*0986 ±*	*0*.*023*	*0*.*3115 ±*	*0*.*026*	*0*.*5164 ±*	*0*.*206*	*5*.*3004 ±*	*0*.*726*
	*MAM-E*	*0*.*2062 ±*	*0*.*037*	*0*.*3649 ±*	*0*.*047*	*1*.*5453 ±*	*0*.*444*	*9*.*9702 ±*	*1*.*625*

### Spatial coherence of CA1 pyramidal cells is impaired in MAM and improved in MAM-E

**Fig 2:**
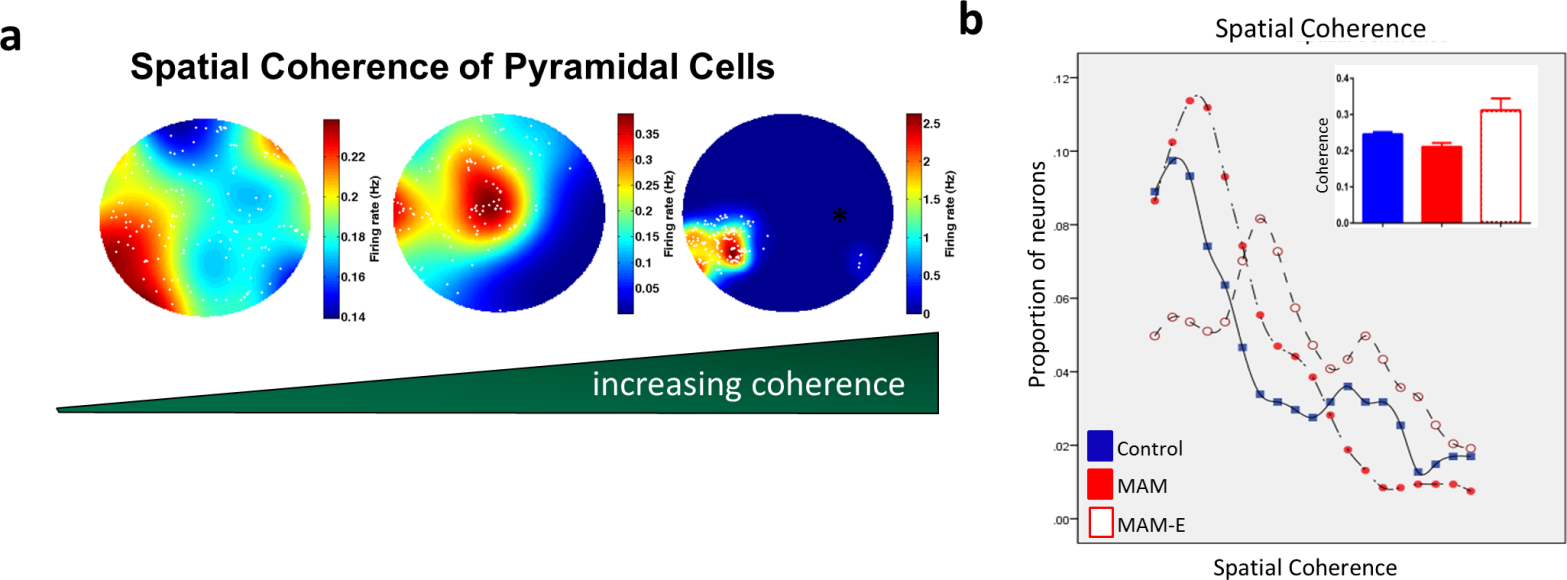
Spatial coherence of neurons from MAM rats is significantly lower than control and MAM-E. Spatial coherence is a good measure of how well a location in space is rate encoded (a); cells with higher coherence (right) have very tight regulation of firing in a specific region of space, whereas cells with lower coherence (left) show more promiscuous firing with very little rate modulation throughout the arena and many out of field action potentials (shown as white dots). Panel (b), shows the spatial coherence values as a moving average of four coherence bins from values of 0 to 0.9 between the groups. This plot illustrates that the coherence distributions are shifted to the right (towards higher coherence values) in the MAM-E group (open red circles) compared to the controls (closed blue circles) or the MAM animals (closed red circles). Control coherence values have a longer and higher right tail, this tail appears to be absent in the MAM group. This is also seen in plots of group average coherence of pyramidal cells (b, inset) in MAM animals (red bars) compared to controls (blue bars); this is improved with enrichment (red checkered bars).

We hypothesized that, consistent with previously observed behavioral impairments, MAM animals have impaired spatial rate coding in CA1 pyramidal neurons and that EE would ameliorate these abnormalities. We used *spatial coherence* [4] to measure the fidelity of spatial encoding by CA1 pyramidal neurons during open field foraging (Fig 2A). Spatial coherence of pyramidal cell firing was significantly altered by MAM and recovered in the enrichment paradigm. Across all recorded pyramidal cells, coherence was 0.192 ± 0.008 in control animals and 0.145 ± 0.015 in MAM animals (p = 0.005, Wald Chi-Square 19.625, Cohen’s d 0.45; control compared to MAM), consistent with our behavioral findings that MAM animals perform poorly in spatial tasks [2]. Environmental enrichment significantly increased spatial coherence of pyramidal cells to 0.256 ± 0.029 (p = 0.001, Wald Chi-Square 8.637, Cohen’s d 0.41; MAM-E compared to MAM unenriched), consistent with our prior findings that EE significantly improves spatial learning and memory. These changes in spatial coherence can be seen best in Fig 2B, which shows the spatial coherence values as a population average (bar graphs, inset) and as a moving average of four coherence bins by group. While the initial peak in the MAM and control groups is similar, the MAM group does not have the right tail seen in the control group, representing neurons with high coherence values. The MAM-E group has these two peaks, although the lower coherence peak is shifted to the right thereby increasing the average coherence of the whole population. Observations noted in the moving average plot can also be seen as a significant increase in the proportion of cells that met criteria for classical place cells (defined as pyramidal cell having a coherence of 0.3 or greater [7]) from 21% in MAM animals to 42% in MAM-E animals, compared to 32% in control animals. These results demonstrate that EE improves the quality of spatial encoding.

### Timing coding is heterogeneous across groups

**Fig 3:**
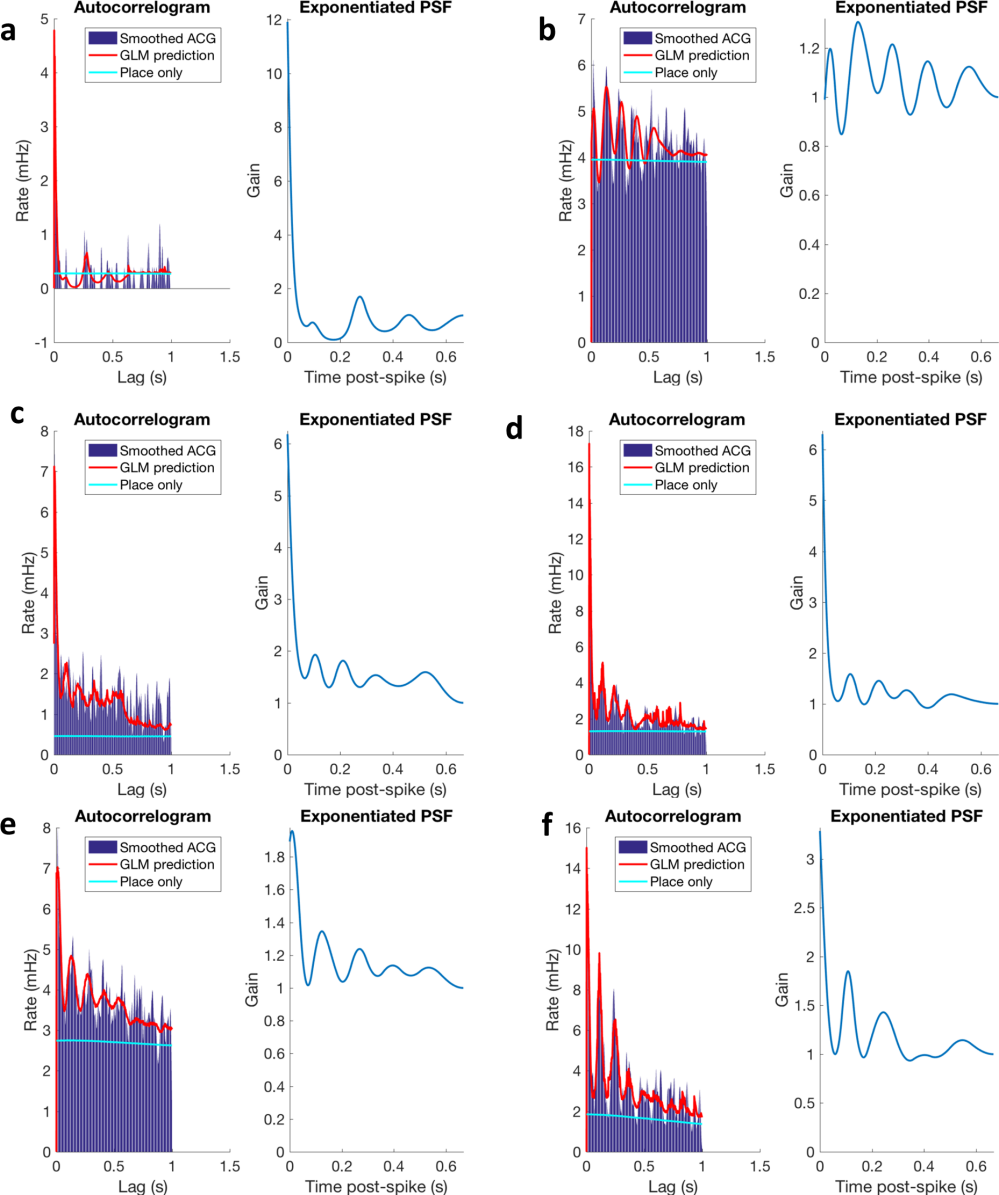
The autocorrelograms (left in each panel) contain oscillatory structure that cannot be modeled with a place field alone (cyan curve). The inclusion of the PSF in the GLM allows the model to accurately predict the autocorrelation structure of neural firing. The exponentiated PSF (right in each panel) corresponds to the time-dependent gain of firing rate. For example, in panel **f** the neuron has an approximately 3.5-fold up-regulation of firing probability immediately after a spike, and an approximately 1.8-fold up-regulation one theta cycle after a spike. These examples were chosen at the 5^th^, 20^th^, 40^th^, 60^th^, 80^th^, and 95^th^ percentiles of the distribution of coherences (low-to-high; panels **a-f** respectively).

The non-random firing of spikes within theta oscillations of the local field potential (LFP) and relative to each other (autocorrelation) is strongly associated with spatial cognition [8]. These phenomena fall under the broad rubric of *timing coding*, and require microcircuits that can dynamically modulate firing probabilities of neurons. Because MAM has significant effects on neural anatomy, we hypothesized that MAM results in damaged hippocampal circuits whose dynamics are suboptimal for encoding spatial information and that EE would improve these dynamics. To quantify the timing coding of neurons, we modeled spike trains using a GLM. Mathematically, our GLM is a statistical model of the firing probability of a neuron that simultaneously incorporates spatial rate coding (the *place field*) and fine spike timing (FST) properties of the cell (Fig 1C). The FST properties include all effects where the relative timing of spikes is different from predicted based on spatial rate coding alone. This means that the autocorrelation of spiking is different from that expected when only a place field is used (in an inhomogeneous Poisson model) to model the spike train (Fig 1C). FST results from the fact that the firing probability of a cell changes as a function of the history of the spike train, i.e. the timing of a previous spike influences the current firing probability (Fig 1C). The GLM captures FST properties using post-spike filters (PSFs), which are parameterized curves that describe the modulation of firing probability after a spike (Fig 1E). The PSF, therefore, is an intermediate functional phenotype describing the high-speed modulation of the probability of spiking. Adding the PSF to the GLM accounts for the high-frequency autocorrelation structure of neural firing (Fig 3). Moreover, the PSF is incorporated in the model over-and-above the rate modulation of the place field (Fig 1C), and can be interpreted as the component of the autocorrelation function that is corrected for spatial rate modulation. This latter property is particularly important in the present study as we seek to directly relate FST parameters *per se* to place field parameters, all of which are blended together in the raw autocorrelation function.

To make comparisons among cells, it was necessary to first normalize all PSFs to have total power equal to one since the *total power* of the PSFs, i.e. the integral of the squared PSF, varied substantially and was strongly correlated to firing rate (see Materials and Methods).

**Fig 4:**
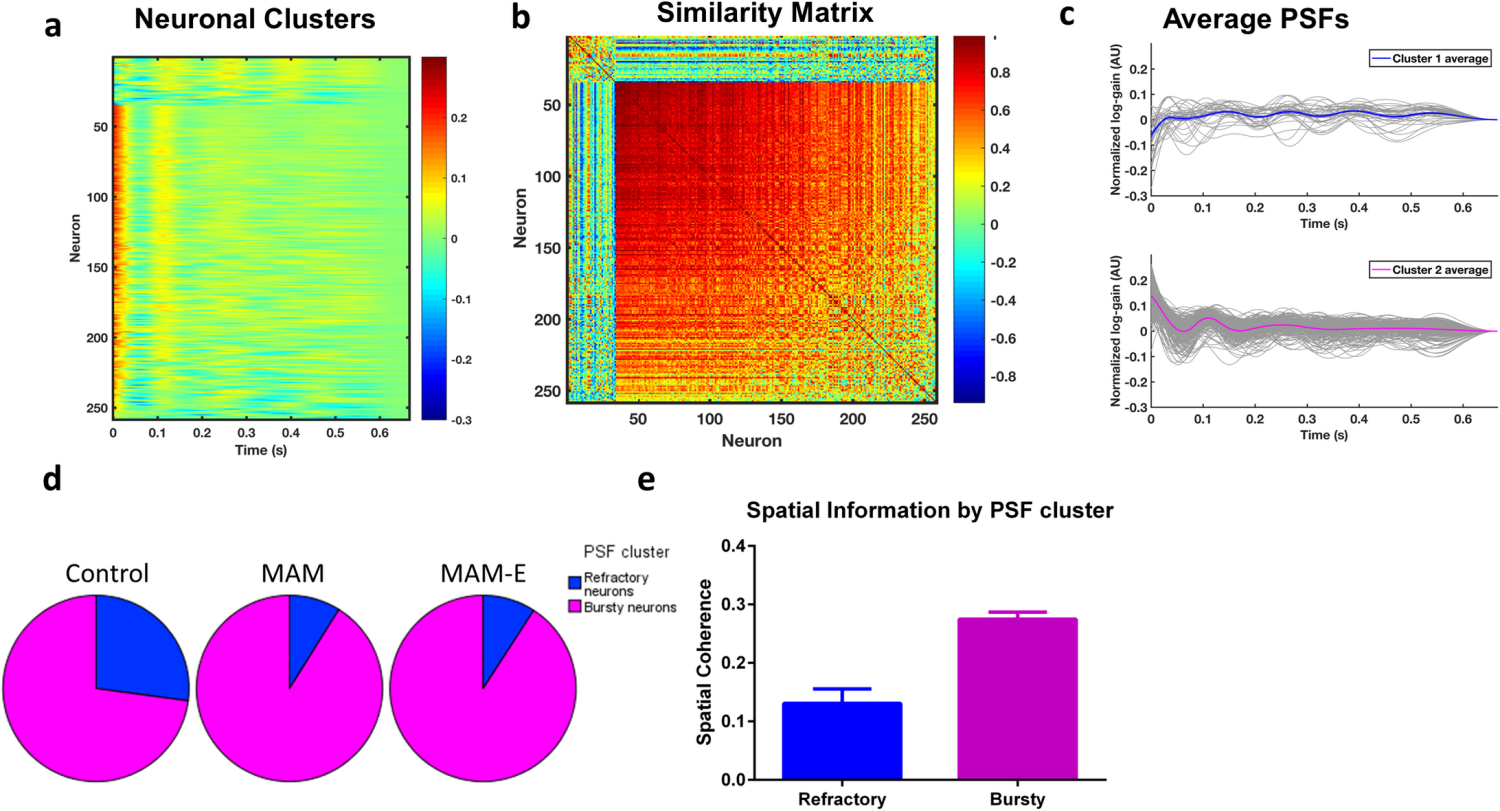
CA1 neurons *in vivo* cluster into two distinct populations, whose distributions differ by group. Neurons from all groups cluster into two main clusters, shown by the heat map of PSFs from the whole population (a) sorted by similarity (see also b). Average traces of the PSF from bursty and refractory neurons show that the probability of a spike in bursty neurons (c; magenta) is rapidly up-regulated in the first 30ms post-spike, followed by down-regulation and subsequent upregulation at 110 ms, approximately one cycle of theta. Refractory neurons do not show this initial upregulation (c; blue). Envelopes around the PSFs represent one standard error; gray traces represent individual PSFs for the entire population (c). Pie charts (d) of bursty (magenta) and refractory (blue) populations shows that the distribution of these neuronal populations differs among the groups, suggesting that post-weaning environmental enrichment does not simply alter the proportion of neurons in the hippocampus with bursty or refractory firing properties. The clusters also contain different amounts of spatial information (e), as shown by average spatial coherence of the different cell types, with bursty cells showing significantly higher average coherence.

To analyze the timing coding properties of each cell, we first performed an exploratory principal component analysis (PCA) on the PSFs (S1 Fig). The coefficients of the PSF time points along the first principal component (PC) show a high peak just after t = 0 and smaller peaks at ~110 ms intervals (i.e. 9 Hz theta oscillations), demonstrating that the dominant variation across the PSFs relates to immediate post-spike and theta modulation. This variation can also be seen in a heatmap of PSFs sorted by their scores along the first PC (S1B Fig). The pairwise correlation matrix of the PSFs with rows and columns sorted by the first PC indicates a dominant cluster of PSFs (S1C Fig; lower right) and a much smaller cluster of PSFs (S1C Fig; upper left) that are anti-correlated with the PSFs in the large cluster. This suggests at least two distinct PSF shapes in the data. To identify these shapes, we clustered the PSFs using the k-means clustering algorithm (k = 2) with correlation distance [9,10]. A heatmap of the PSFs sorted by cluster shows marked differences in the post-spike timing modulation (Fig 4A), and the correlation matrix sorted by cluster shows high within cluster correlations and low out-of-cluster correlations (Fig 4B). As expected, the clusters also differ significantly in their scores along the first PC (S1D Fig).

The first, and smaller, cluster contained cells (*refractory cells*; Fig 4C, top) whose firing was slightly down-regulated immediately following a spike (negative value of PSF immediately after t = 0, Table 2). The second, and larger, cluster contained cells that were markedly up-regulated post-spike (*bursty cells*; Fig 4C, bottom). Bursty and refractory cells did not differ in half width (p = 0.87, Wald Chi-Square 0.705), but did differ in several different firing parameters (Table 1). Refractory neurons had a higher mean firing rate (p = 0.035, Wald Chi-Square 4.429, Cohen’s d 5.00), and a lower instantaneous firing frequency (p<0.001, Wald Chi-Square 228.706, Cohen’s d 0.95). The bursty cells also showed consistent theta modulation, where the probability of firing was up-regulated 1 theta cycle (~110 ms) after a spike (Fig 4A and 4C). The first cluster showed suggestive theta modulation at a slower frequency. Measures of burstiness (immediate post-spike integral) and theta modulation are summarized by cluster and by group in Table 2. Significant cluster effects were found for immediate post-spike integral (p<0.001, Wald Chi-Square 216.983, Cohen’s d 2.70), theta peak time (p<0.001, Wald Chi-Square 55.768, Cohen’s d 1.05) and theta trough (p = 0.007, Wald Chi-Square 7.182, Cohen’s d 0.05). No cluster effect was found for theta correlation or theta peak (p = 0.34 and p = 0.29, respectively).The relative proportions of these two clusters of cells differed by group (Fig 4D), with control animals having significantly more refractory cells than either MAM or MAM-E: 6.2% of cells in controls vs 3.5% in both MAM and MAM-E animals.

**Table 2:** Description of the PSF parameters of all recorded populations of pyramidal neurons. The table breaks characteristics down by cell firing properties (bursty cells in normal-type font the top three rows, refractory cells italicized in the bottom three rows) and by group (Control shaded in blue, MAM in red, MAM-E in beige).

		Immediate Post-Spike Interval Value			Theta Correlation			Theta Peak			Theta Peak Time			Theta Depth			Theta Trough			Theta Integral		
Bursty	Control	3.01E-03	±	1.98E-04	3.64E-01	±	1.78E-02	5.72E-02	±	2.29E-03	1.26E-01	±	2.36E-03	1.27E-01	±	9.87E-03	-1.39E-02	±	9.09E-03	7.34E-02	±	6.55E-02
	MAM	3.13E-03	±	1.63E-04	3.64E-01	±	1.95E-03	5.21E-02	±	6.12E-04	1.32E-01	±	4.13E-03	1.26E-01	±	8.30E-04	-1.40E-02	±	4.44E-04	1.07E-01	±	1.00E-01
	MAM-E	3.37E-03	±	1.83E-04	3.84E-01	±	1.78E-03	5.72E-02	±	8.42E-04	1.38E-01	±	4.96E-03	1.36E-01	±	1.58E-03	-1.89E-02	±	1.71E-03	3.90E-01	±	5.62E-02
*Refractory*	*Control*	*8*.*61E-04*	*±*	*2*.*87E-04*	*3*.*38E-01*	*±*	*4*.*13E-02*	*3*.*72E-02*	*±*	*5*.*53E-03*	*1*.*13E-01*	*±*	*1*.*27E-03*	*1*.*68E-01*	*±*	*6*.*96E-03*	*-4*.*18E-02*	*±*	*5*.*19E-03*	*2*.*43E-01*	*±*	*2*.*67E-02*
	*MAM*	*1*.*06E-03*	*±*	*1*.*16E-04*	*3*.*77E-01*	*±*	*2*.*79E-02*	*4*.*36E-02*	*±*	*8*.*88E-03*	*1*.*13E-01*	*±*	*9*.*95E-04*	*1*.*69E-01*	*±*	*1*.*82E-02*	*-3*.*76E-02*	*±*	*1*.*49E-02*	*2*.*04E-01*	*±*	*6*.*87E-03*
	*MAM-E*	*1*.*46E-03*	*±*	*8*.*78E-05*	*3*.*22E-01*	*±*	*4*.*42E-02*	*7*.*24E-02*	*±*	*3*.*08E-03*	*1*.*17E-01*	*±*	*7*.*49E-04*	*1*.*57E-01*	*±*	*1*.*12E-02*	*-1*.*97E-02*	*±*	*7*.*43E-03*	*2*.*72E-01*	*±*	*1*.*52E-02*

The spatial coherence of the cells was dramatically different between the clusters (Fig 4E). The bursty cells had significantly higher coherence than the other cells (0.27 ± 0.012 vs. 0.13 ± 0.027, *p* < 0.0001, Wald Chi-Square 23.038, Cohen’s d 0.78). Because of the critical differences in timing and cognitive content of the bursty cells, we retained only the bursty cells for further analysis.

**Fig 5:**
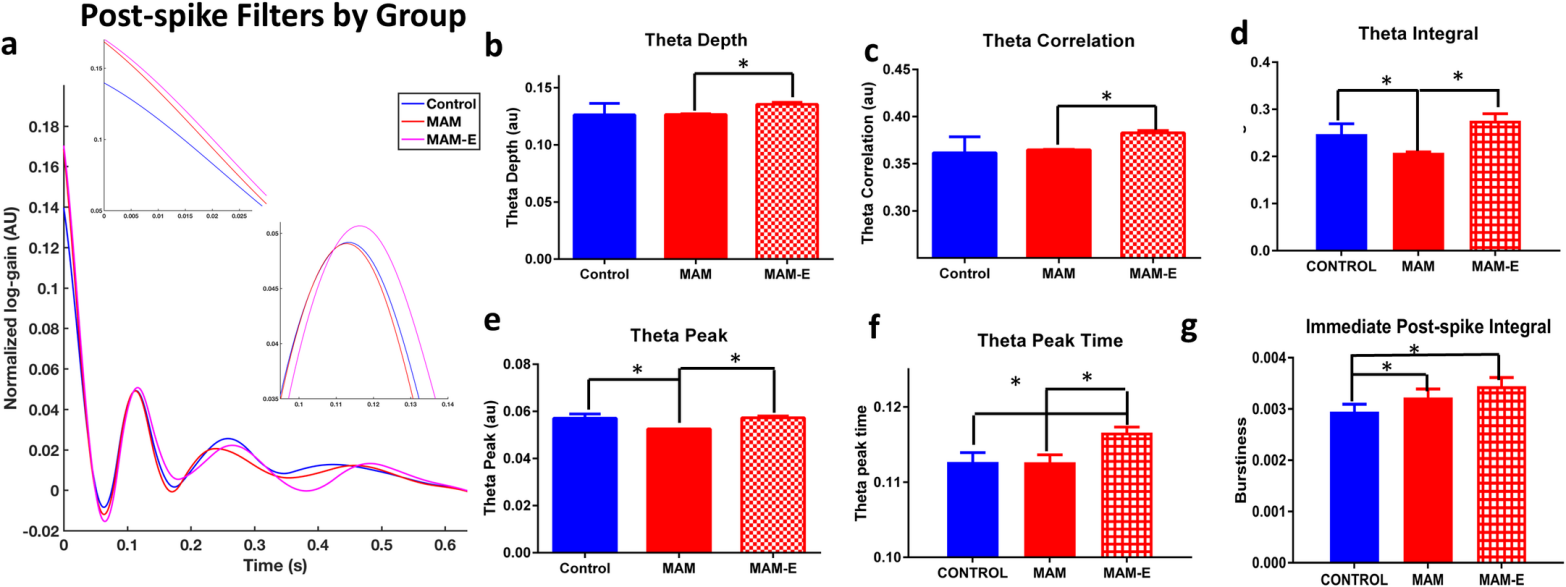
PSF parameters are altered in MAM animals and modified by environmental enrichment. Average post-spike filters by group (a) show clear differences in the shape of the filters by group, particularly at the initial ms post-spike, and at timepoints 0.1s post-spike and 0.2s post-spike, corresponding to the first and second phase of theta. MAM neurons (red filled bars) reduced theta depth (b) and reduced theta correlation (c) compared to MAM-E animals (red checkered bars). MAM neurons also show reduced theta integral (d) and theta peak (e) compared to both MAM-E and control animals (blue bars). MAM-E neurons show an increase in theta peak time (f) compared to both control and MAM neurons. MAM and MAM-E neurons both show increased burstiness (g) compared to controls.

The PSFs of bursty cells differed between groups (Fig 5A). The MAM and MAM-E cells have significantly higher up-regulation immediately post-spike (p = 0.009, Wald Chi-Square 6.854, Cohen’s d 0.28 control compared to MAM; p<0.0001, Wald Chi-Square 13.396, 0.50 control compared to MAM-E), while the MAM-E and control cells had stronger up-regulation one theta cycle after a spike. Controls cells showed the strongest theta modulation, with significant up-regulation even at the second theta cycle post-spike.

### MAM animals exhibit altered timing coding that is improved by EE

To quantify differences in bursty PSFs among groups, we parameterized the bursty PSFs by key features that captured the significant quantitative variation across this subset of cells, namely immediate post-spike modulation and theta frequency modulation (S1 Fig). The first parameters we examined were modulation of firing in the theta frequency range (6–12 Hz). We measured theta modulation in several ways (see Materials and Methods): up-regulation one theta cycle post-spike (*theta peak*), the time of the theta peak (*theta peak time*), down-regulation between a spike at t = 0 and one theta cycle later (*theta trough*), the difference between theta peak and theta trough (*theta depth*), the correlation of the PSF to a theta-frequency sinusoid (*theta correlation*), and the integral of the PSF under the “theta bump”, i.e. the cumulative up-regulation of the PSF in the interval 83 ms to 250 ms post spike (*theta integral*). Neurons from MAM animals have less precise theta modulation (Fig 5B–5F) with a higher theta trough (p<0.001, Wald Chi-Square 22.283, Cohen’s d 0.11), lower theta correlation (p<0.001, Wald Chi-Square 46.478, Cohen’s d 0.15), lower theta depth (p = 0.01, Wald Chi-Square 6.521, Cohen’s d 0.21), lower theta peak (p<0.001, Wald Chi-Square 48.792, Cohen’s d 0.24), lower theta integral (p<0.001, Wald Chi-square 23.126, Cohen’s d 0.30) and a shorter theta peak time (p = 0.001, Wald Chi-Square 13.152, Cohen’s d 0.27) when compared to MAM-E animals (see Table 2 for details).

In addition to modulation within the theta frequency range, we observed differences in the up-regulation of firing in MAM animals immediately after a spike. We defined the *immediate post-spike integral* (*burstiness*) of a PSF as the integral of the normalized PSF over the interval 0–30 ms (Fig 5G). This captures the strength of up-regulation immediately after a spike, i.e. increased burstiness. Consistent with previous in-vitro experiments showing increased bursting and hyperexcitability in MAM animals [11] both MAM and MAM-E animals have increased burstiness compared to controls (0.0029 ± 0.0001 in controls vs 0.0033 ± 0.0001 in MAM and 0.004 ± 0.0001 in MAM-E, *p* < 0.01, Cohen’s d 0.28 control vs MAM, Cohen’s d 0.50 control vs MAM-E, Cohen’s d 0.22 MAM vs MAM-E, Wald Chi-Square 15.455 for group; Fig 5F). The significant difference between MAM-E and control cells in this initial interval suggests that some timing coding changes persist in malformed brains, even after rescue of behavioral and cognitive phenotypes.

### Variation in fine spike timing predicts spatial coherence

The PSFs measure timing properties of neuronal firing while spatial coherence measures the quality of the neural encoding of external space. The former measures action potential timing *per* se, while the latter measures the match between the outside world and its internal representation. These phenomena are strongly linked; alterations in timing coding are related to cognitive impairment in control animals and disease models. To initially explore the relationship between the PSFs and spatial coherence in MAM animals, we correlated each PSF time point to spatial coherence (S2A Fig). As with the differences between groups, two distinct time periods distinguished themselves (S2A Fig): the immediate post-spike interval (17 ms, peak C = 0.39) and one theta cycle post-spike (121 ms, peak C = 0.54). There were weaker correlation peaks at later theta cycles. This suggests that the dominant predictors of coherence within the PSFs relate to these two time intervals. A similar analysis using the smoothed autocorrelograms (ACGs) for each neuron shows a high correlation (> 0.5) between every time point and spatial coherence (S2B Fig), with little variation across the time interval. This demonstrates that the raw ACG can be used to predict coherence, but this prediction is dominated by slow variation on behavioral timescales, not circuit-level time scales measured by the PSF.

**Fig 6:**
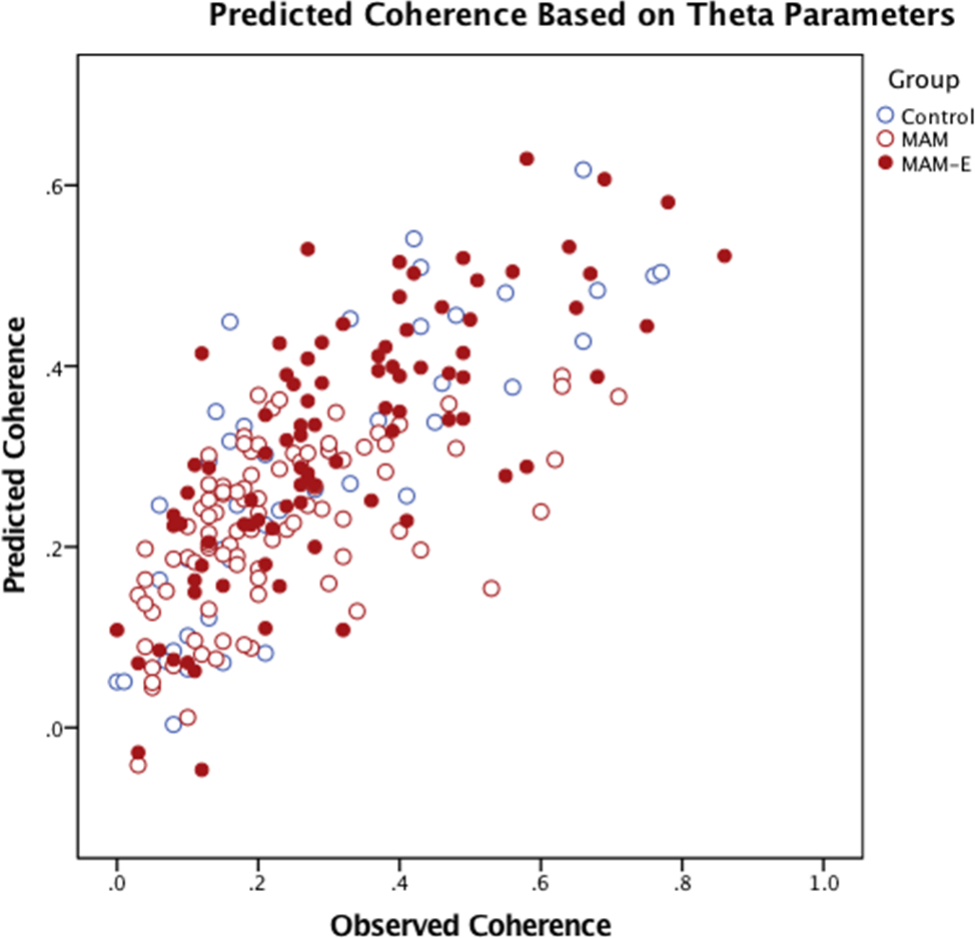
PSFs predict spatial coherence. These measures of fine-spike timing do not, necessarily, have to correlate to spatially-modulated rate coding of pyramidal neurons in the hippocampus; however predicted coherence, obtained from regression analyses of the PSF parameters against coherence, plotted against actual coherence shows a clear positive relationship (c; R^2^ = 0.617) between PSF parameters and spatial coherence in all groups. This confirms that the PSF parameters predict spatial coherence.

Variation in the above PSF parameters that stratify groups also predict spatial coherence, suggesting a direct link between local circuit dynamics and the construction of internal representations of the external world in control and MAM animals. Group, initial value, theta depth, theta correlation, theta peak, theta peak time, theta trough and theta integral were correlated with coherence in a multivariate regression that included the main effects and all two-way interactions. Remarkably, the PSF parameters accounted for 61.7% of the variance in spatial coherence in this regression (Spearman’s r^2^ = 0.617, p<0.001; Fig 6), suggesting that the FST of the CA1 pyramidal cell firing is directly related to place-modulated rate coding of these neurons. Furthermore, significant effect of group in the model is eliminated when PSF parameters are modeled, suggesting that much of the group differences in spatial coherence is a function of theta modulation of neuronal firing, thus further supporting the view that modification of cell firing in the theta frequency could improve cognitive outcome.

**Fig 7:**
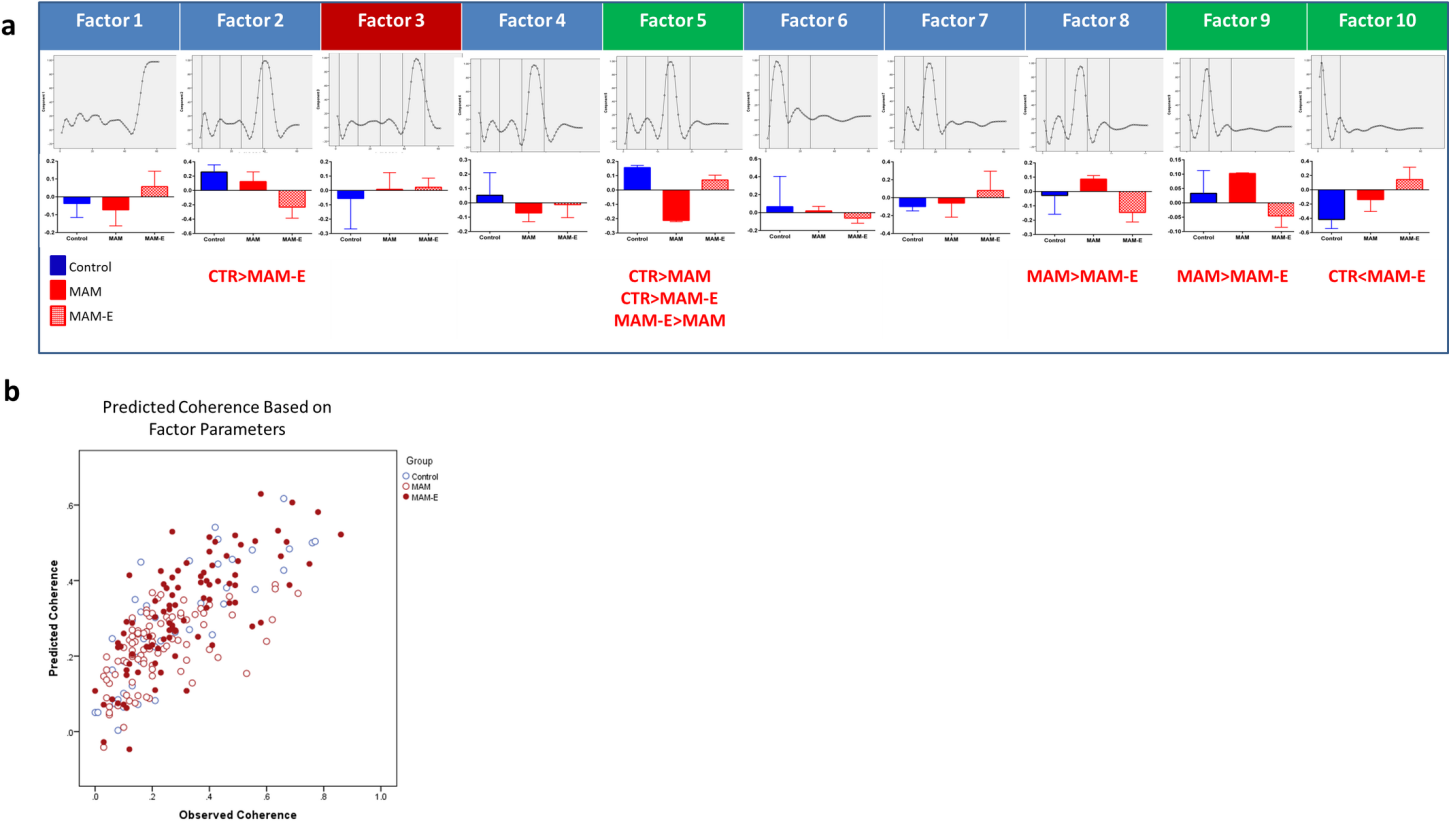
PSF factors are altered in MAM animals and modified by environmental enrichment. **Factors also predict spatial coherence.** A PCA divides the PSF into 10 factors, shown as loading plots for each of the individual factors in the top row. Shown in a, Vertical lines represent the initial value time point (first line) and successive oscillatory cycles of theta (second-fourth lines). Factors 3, 5, 9 and 10 significantly predict spatial coherence; factors 5, 9 and 10 positively predict coherence (green label) while factor 3 negatively predicts coherence (red label; p>0.001). Factors 2, 5, 8, 9 and 10 show group differences. Differences between MAM-E and control cells in factor 2 suggest that MAM-E cells are less modulated at the third theta peak; however, this factor is not significantly related to spatial coherence. Differences between MAM and MAM-E cells in factor 8 suggest that there is more modulation in between theta peaks in MAM cells; however, this factor is also not significantly related to spatial coherence. Factors 5, 9 and 10 relate to spatial coherence and show group differences as well. These factors show strong modulation at the second (factor 5) and first (factor 9) theta peaks, as well as at the initial post-spike interval region (factor 10), thus corroborating the importance of fine spike timing in these time periods. Factor scores also significantly positively predict spatial coherence as well (b; R^2^ = 0.753).

To corroborate the above findings, and determine whether we identified a comprehensive set of timing parameters, we performed PCA on the set of bursty PSFs. Because we used a 10-dimensional basis for the PSFs (see Materials and Methods) there were ten factors that explained 100% of the variance in the PSFs (Fig 7A). Using these 10 factors as inputs into a multivariable regression (including pairwise interaction terms), we found that three factors were significantly associated with coherence (true vs. predicted R^2^ = 0.753; Fig 7B). A table of total variance explained by factor (S4 Fig) shows that no particular factor explains all of the variance in the model. These factors had strong loadings in the interval just after the spike, and at the first and second theta cycles post-spike (Fig 7A), demonstrating as in our exploratory analysis that the critical time points for predicting coherence align with the immediate post-spike interval and the peak of each subsequent theta cycle. Moreover, the scores for each of these factors differed among the experimental groups. The 10 factors were compared across groups and validate the findings from analyses of the *a priori* defined parameters of the PSF (Fig 7A). Importantly, the significant factors that corresponded to theta modulation were higher in controls and MAM-E, whist the significant factor that corresponded to out of theta firing was higher in MAM animals, further supporting the view that MAM animals have disrupted timing coding. This indicates that these time points are differentially regulated among the groups.

In order to ensure these results weren’t due entirely to overfitting given the number of regressors used in our model and the number of cells included, we paired down the number of variables included in our models. In the first regression analysis we had included theta peak, theta peak time, theta trough, theta correlation, theta integral and initial value, with all two-way correlations. To reduce the number of parameters and thus minimize overfitting we carried out a factor analysis (see below) including all of the above parameters. Two orthogonal factors were extracted and included in a regression model. In this reduced model the spearman r^2^ value of the correlation between predicted versus observed spatial coherence is 0.551, similar to the 0.617 reported above. If only group is included the correlation is 0.24.

For the second regression we reduced the number of parameters in the model by excluding all 2-way interactions and only retaining the 4 significant main effects (Factors 3,5,9 and 10). In this situation the spearman r^2^ for the correlation between observed and predicted spatial coherence was 0.56, compared to 0.753 reported. These analyses together suggest that between 56% and 75% of the variance is explained by our measured parameters

### EE is associated with a partial rescue of parvalbumin-positive interneurons in CA1

**Fig 8:**
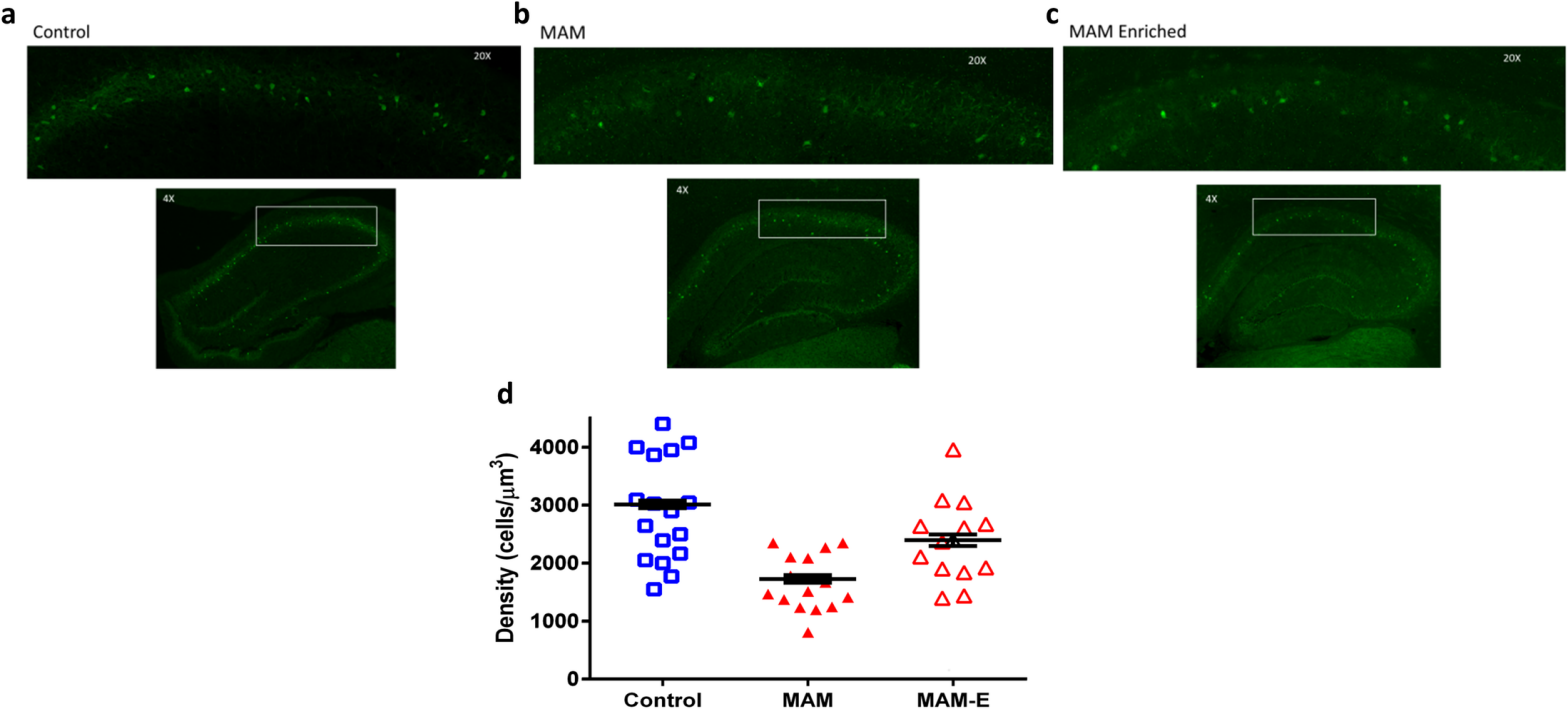
Environmental enrichment normalizes PV^+^ interneuron numbers. Immunohistochemistry for parvalbumin positive interneurons at 20x (top panel) and 4x (bottom panel) magnification in (a) control animals, (b) MAM animals and (c) MAM-E animals. Quantification (d) in MAM animals (red closed triangles) show a stark decrease in the number of PV^+^ interneurons per μm^3^ in CA1 compared to their control counterparts (blue squares). Environmental enrichment (red open triangles) is able to significantly increase the numbers of PV^+^ interneurons in CA1.

Interneurons, particularly somatic and axon initial segment-targeting interneurons, are crucial for timing of pyramidal cell outputs [12]. This subset of interneurons often expresses the calcium-binding protein marker parvalbumin (PV). Given the aberrant temporal coding in MAM animals and restoration MAM-E animals, we examined counts of PV^+^ neurons post-mortem. We found a stark reduction in PV^+^ neurons in MAM adult animals from 4154.89 ± 91.05 cells/10,000 μm^3^ in controls to 1747.23 ± 62.58 cells/10,000 μm^3^ (p<0.0001, Cohen’s d 1.63; Fig 8). Enrichment attenuated this decrease, with MAM-E animals having 2363.35 ± 219.49 cells/10,000 μm^3^ (p = 0.002 compared to MAM, Cohen’s d 0.42; Fig 8). Given the importance of PV interneurons for temporal organization of pyramidal neuron firing, we suggest that this may represent a major cellular mechanism by which enrichment improves temporal structure.

## Discussion

Our results definitively show that rats with MAM-induced malformations of cortical development have disruptions to hippocampal space and timing coding that likely underpin previously identified impairments in spatial cognition. Despite developing with a significant structural brain abnormality, exposure of MAM rats to post-weaning environmental enrichment improves several characteristics of the rhythmic dynamics towards normal, consistent with our previous behavioral observations. Importantly, we also observed a clear relationship, both across and within groups, between the timing fidelity of action potential firing, as measured with a post-spike filter, and the spatial fidelity as measured by spatial coherence. Environmental enrichment is the only therapeutic technique that has been shown to improve cognition in any model of MCDs [2], so it presents a critical window into the functional changes that occur in response to successful treatment. In this way, our data suggest that disrupted rhythmic dynamics can be conceptualized as a system-level mechanism of cognitive impairment. Therefore, therapies that directly target rhythmic dynamics have enormous promise for ultimately improving cognitive outcomes in patients with malformations of cortical development.

The hippocampus is a crucial brain structure supporting cognition and is a site of massive integration of information. Healthy brain function requires neural activity to be precisely timed, allowing effective coordination within and between brain regions [8]. When the hippocampus is damaged or malformed there are a huge number of alterations to brain physiology, including: changes in neuron number [13], synaptic function [14], a variety of cell signaling pathways including neurotrophic pathways [15] and neurotransmission [16,17]. All of these alterations could potentially contribute to impaired cognition, either independently or in concert. Restoring cognition post-insult requires modifying the pathological dynamics of the impaired neural network. To this point, it should be noted that individual effects of each of the PSF parameters is small, but altogether in a regression these account for a significant proportion of the variance between the groups. This further supports the fact that pathological differences in FST are not in one particular parameter of the FST but rather about general temporal modulation of firing dynamics.

In the case of MCDs, the brain must generate sufficiently normal-like dynamics using an abnormal neural substrate. Identifying therapeutic targets to induce such changes in dynamics is critical for developing therapies for improving cognitive outcomes for patients with MCDs. These targets need to capture the function of the full neural network as a complex system, and necessarily exist at an abstract layer above all of the genetic, molecular, and cellular processes that are critical to maintain a living neural network. Our results are a significant step in this direction. We show that the encoding of external spatial information, the cognitive representation, is strongly related to the timing properties of firing, and that this quantitative relationship persists across control animals, MAM animals, and MAM animals that received EE. In other words, timing coding predicts cognitive encoding quality across normal and abnormal brains, and in the presence or absence of cognitive impairment. The relationship to disease here is important. It is now well established that under control conditions the timing of action potentials from CA1 pyramidal cells is spatially structured over-and-above spatial rate coding, e.g. through phase fields [18,19]. Likewise, alterations in phase coding have been reported in disease models [20]. *A priori* these facts do not necessarily relate to each other. This study shows that the relationship between timing and rate coding that exists in controls accounts for the significant differences in spatial coherence between control, MAM, and MAM-E animals. Rather than being categorically different in their encodings, MAM animals appear to simply be poorly tuned, and EE appears to improve much of this poor tuning. We posit, therefore, that timing coding itself is a systems-level target for improving cognitive outcome in MCDs.

The MAM model is used to model an etiology that is often associated with intractable pediatric epilepsy in patients, but that does not produce frequent seizures in rodents, allowing us to understand the effects of etiology independently of seizures. However, *in utero* insults and cortical malformations are common to many neurodevelopmental disorders [21–23]. Therefore, understanding how abnormal neural networks can functionally reorganize to support optimal cognition may have far-reaching implications. In prior work, we have shown that EE in MAM animals leads to cognitive gains [2]. In the present work, we take an important step further to show that EE improves multiple features of timing coding in a subpopulation of neurons whose firing resembles previously-reported complex spiking neurons [24], and that these alterations predict improved cognitive encodings. Moreover, this improvement with EE is concurrent with the preservation of at least one class of neurons (PV^+^ interneurons) that primarily control the spike timing of CA1 pyramidal cells through dynamically modulated somatic inhibition [12,25]. Although this alteration in neuron number is potentially compelling, we recognize that our sample sizes are relatively small and our histology was conducted not only in the area proximal to the recording site but through a large swath of the dorsal/ventral axis of the hippocampus, where there could be potential differences in development of this axis in MAM animals could confound our interpretation of the results; therefore this result needs to be confirmed by future studies and followed up by a more extensive characterization. Nevertheless, while we do not believe that increases in PV^+^ interneuron numbers are the only relevant histological change in MAM-E brains, there is a well-established relationship between these neurons and the theta oscillatory behavior of CA1 pyramidal cells [12], we believe this makes them a highly likely component of the substrate producing the systems-level changes we observe in spike timing. It is important to note however, that the hippocampal networks in MAM animals developed around a context in which there are fewer PV interneurons. There is likely compensation that has developed in order to maintain homeostasis, although this compensation is not restoring the system to normal and thus these animals have heightened seizure susceptibility and cognitive impairment [26–28]. This is distinctly different to acute modulation of PV interneurons in a control brain [29] and thus restoration of PV interneuron numbers that occurs with EE in MAM animals should be interpreted in the context of a chronic alteration in inhibition within the network.

The direct relationship between timing and cognition that we found spanning both a disease model (MAM) and a therapeutic rescue model (MAM-E) suggests that modifying rhythmic dynamics is critical for restoring cognition. Our results are thus complementary to important recent work showing that driving neural circuits at specific frequencies can result in cognitive gains in patients with Alzheimer’s disease and other diseases [30]. Our results suggest the development of therapies that directly intervene in the timing coding of cells. The advent of sophisticated electrical and optogenetic stimulation approaches, along with cell-based therapies such as interneuron precursor implantation, makes such strategies potentially viable.

## Materials and methods

### Animals

All animal procedures were approved by the Dartmouth College Institutional Animal Care and Use Committee and University of Vermont Institutional Animal Care and Use Committee, under United States Department of Agricultural and Association for the Assessment and Accreditation of Laboratory Animal Care International approved conditions, in accordance with National Institutes of Health guidelines. Sprague-Dawley rats housed with a 12 h light/dark cycle and *ad libitum* access to food and water.

*MAM model*: Dams were randomly selected for intraperitoneal injection with either 20 mg/kg MAM (Midwest Research Institute Global, Kansas City, MO) or saline at embryonic day (E) 17.

### Enrichment paradigm

Pups were raised with their dams until weaning at postnatal day (P) 25, when they were then randomized to either standard or enriched housing until electrophysiological studies were performed (>p70). Males and females were included. Animals that were enriched were housed in breeder cages (38 x 48 cm) with 1 to 3 other animals as well as various colorful and differently shaped and textured inanimate objects. Objects were rotated once per week. Animals that underwent standard care were housed singly in a rat cage with no inanimate objects. All animals stayed in their assigned environment throughout the behavioral experiments and underwent only the reported treatments, behavioral and electrophysiological experiments until they were sacrificed for histology. Due to the nature of the experimental setup, experimenters could not be blinded to group. 15 rats were used: 4 controls (59 cells), 7 MAM unenriched (101 cells), and 4 MAM-E (98 cells).

### Surgical implantation of electrodes

Rats were anesthetized with isoflurane (2–3% in oxygen) and placed in a stereotaxic frame (Kopf Instruments, Tujunga, CA). Custom-built electrodes containing four independently drivable tetrodes were implanted 3.2 mm posterior to bregma, 2.2 mm lateral and 1.7 mm deep from skull surface into the dorsal CA1 hippocampus. MAM coordinates were adjusted to account for 10% reduction in total brain size based on previous MRI measurements of MAM animals [2]; coordinates were 2.9 mm posterior to bregma, 2.0 mm lateral to midline and 1.5 mm ventral to skull surface. Electrode placement was verified visually post-mortem, although data loss occurred that precludes the inclusion of images of the electrode track in this manuscript.

### Data acquisition

Tetrode assemblies were advanced 50 μm twice a day until hippocampal theta oscillations (6–12 Hz), sharp waves and ripples were observed in the EEG. Electrodes were then advanced in 25 μm increments until CA1 single unit activity was detectable. Single unit activity was recorded when waveforms above 40 μV in amplitude were observed on one or more tetrodes. The signal from the electrodes was preamplified directly from the rat's head by operational amplifiers and transmitted via a custom cable to a Neuralynx recording system (Neuralynx, Bozeman, MT).

Recordings were performed in an open arena. The arena had a diameter of 75 cm and the walls and floor were made of plywood and painted grey. To provide a visual anchoring cue, a white piece of paper covering 100° of arc was attached to the wall of the arena. Rats were allowed to freely explore the arena for 15 min per session per day. To avoid recording the same cells twice, the tetrodes were advanced at the end of each session. The animals were tracked in concurrent video recordings so that cell firing relative to position could be analyzed.

### Electrophysiological analysis

Action potentials were clustered using Neuralynx Spike Sort 3D software (Bozeman, MT). Once cells had been clustered, firing rate maps were computed in MatLab (MathWorks, Natick, MA) showing action potential firing as a function of position. We calculated place fields by smoothing spatially binned spike counts and occupancy maps (time spent in each pixel) using a 2D Gaussian kernel. We chose the width of the kernel using 5-fold cross-validation with the log-likelihood of the resulting place field model as the measure of model fit. Due to the recording protocols, very few interneurons were recorded over all. Those that were recorded were identified as distinct from pyramidal cell groups in that they typically do not exhibit place preference and have a much higher rate of firing than pyramidal cells. Interneurons or neurons that did not appear to be hippocampal in origin were excluded from further analysis. PSFs from the one animal excluded on the basis of having only non-hippocampal neurons recorded are shown in S3 Fig.

Spatial coherence of pyramidal cells was calculated for the entire population as previously described. Coherence is defined as the correlation (2D z-correlation) between the rate in each pixel and the average rate of its 8 nearest neighbors [20]. The peak rate of a field corresponded to the average rate in the highest firing pixel and its neighbors. Most analyses were conducted on the entire pyramidal cell population; for comparison purposes, “classical place cells” were defined as pyramidal cells with a spatial coherence of 0.3 or greater, as previously reported [20]. Inclusion of a given recording session required adequate sampling of the environment and place cell coherence was evaluated based on speed-filtered sessions where the animal was traveling 5 cm/s or faster. Mean firing rate was calculated as the number of action potentials divided by the total time of the recording session. Instantaneous firing rate was calculated as 1/peak interspike interval. Spike width was calculated by locating the first point and the peak of the waveform, and then finding a point that was 25% maximal height and calculating the time between the two measures.

In addition, we computed L-ratio and isolation distance using SpikeSort3D for our entire population of neurons and can show no differences between the groups (p = 0.16 for isolation distance, p = 0.42 for L-ratio), nor are there differences between bursty and refractory neurons (p = 0.41 for the isolation distance, p = 0.79 for the L-ratio). We therefore conclude that our findings are not the result of poorer isolation of neurons in any particular group nor are the two subpopulations of neurons due to a clustering artifact.

### Histology

102 sections from 8 rats: 2 control (4–17 sections), 4 MAM (5–15 sections), 2 MAM-E (8–13 sections) were used. Rats utilized for histological experiments were from a separate cohort than animals used for electrophysiology, so direct comparison between these experiments is not possible. Animals were deeply anesthetized with isoflurane (4–5% in oxygen) immediately after which rats were perfused transcardially with 1x PBS followed by 4% paraformaldehyde (Sigma Aldrich, St. Louis, MO). Brains were postfixed in 4% paraformaldehyde overnight, 30% sucrose embedded until they sank and frozen in OCT tissue embedding solution. Coronal sections (40 μm) through the entire extent of the hippocampus were collected using a cryostat (Leica CM3050, Leica Microsystems). Every fourth section was collected and stained throughout such that a representative swath of the hippocampus was examined. Before immunostaining, sections were washed 2 times in PBS for 15 minutes. They were then placed in a blocking buffer solution composed of 1x PBS, 0.5% Triton, and 10% normal goat serum for 1 hour at room temperature. After blocking, sections were incubated in mouse anti-parvalbumin primary antibody (1:500, Millipore cat # MAB1572, RRID: AB_2174013) for 18 hours at 4°C. Sections were once again washed twice in PBS for 15 min per wash. They were then incubated in species-specific secondary antibody (AlexaFluor 488, Life Technologies) overnight, again at 4°C. After two 10 minute washes in PBS, sections were lastly incubated in DAPI solution for an additional 10 minutes. Sections were mounted on charged slides (Superfrost Plus, Fischerbrand) and coverslipped with mounting medium (Fluoromount, Sigma). We used the optical fractionator function of Stereo Investigator (MBF Bioscience, Williston, VT, USA) to obtain PV numbers. The optical fraction was set at 10 μm depth, centered in the middle of the tissue section encompassed by the counting frame. The optical fraction was realigned for every new counting frame. Only cells falling within the fraction were counted. Cells were counted blindly to reduce subjective bias. To eliminate bias due to area and thickness of tissue, cell counts were divided by volume of the section to obtain the more representative measure of cell density.

### Generalized linear models

We binned pyramidal cell spike trains into Δ =1 ms bins and fit generalized linear models (GLMs) to the resulting binary sequence. The GLM had two time-varying components: the place field of the pyramidal cell, *p*(*x*,*y*), where (x,y) is the 2D position of the rat, and the post-spike filter (PSF), *f*(*s*), which models the post-spike rate modulation as a function of the time, s, after a spike.

For the PSFs, we used ten ‘raised cosine bump’ basis functions, *b*_*i*_(*s*) (i = 1,…,10), extending over 0.7 s and one 1 ms impulse, *b*_11_(*s*), immediately post-spike to capture the absolute refractory period after a spike (cf.[31]). Thus, the PSF is defined by a set of 11 coefficients, *β*, for these 11 basis functions:

$$f_{\beta }(s)=\underset{smooth}{\underset{︸}{\sum_{i=1}^{10}\beta_{i}b_{i}(s)}}+\beta_{11}b_{11}(s)$$

where we call the first term on the left hand side corresponding to the raised cosine basis the *smooth component* of the PSF. The conditional intensity function, *λ*(*t*), of the full model at a given choice of *β* is given by

$$\lambda (t;\alpha_{0},\alpha_{1},\beta )=\exp(\alpha_{0}+\alpha_{1}p\left(x(t),y(t)\right)+\sum_{\tau =1}^{T}r(\tau )f_{\beta }(\tau -t)d\tau )t$$

where *r*(*t*) denotes the spike train, *α*_0_ controls the baseline firing rate of the neuron, *α*_1_ modifies the firing intensity of the place field, *f*_*β*_ is the PSF defined by *β*, and T denotes the length of the recording.

We fit the GLM by maximum likelihood with a ridge penalty. The penalized likelihood function is

$$L\left(\alpha_{0},\alpha_{1},\beta \right)=\sum_{t=1}^{T}\left[r(t)\log\left(\lambda (t;\alpha_{0},\alpha_{1},\beta )\Delta \right)-\lambda (t;\alpha_{0},\alpha_{1},\beta )\Delta \right]-\xi {\left\|\beta \right\|}^{2}$$

where *ξ* is the penalty hyperparameter, which corresponds to the variance of a Gaussian prior on the PSF coefficients. We selected *ξ* from a grid by evidence maximization as in [32]. Note that we only penalize the *β* coefficients, treating the place field and background firing as covariates.

*Clustering post-spike filters*: We normalized the PSFs to have a total power of one and clustered the normalized PSFs using k-means clustering (k = 2). We chose k = 2 by visual inspection of the clustered correlation matrices, and the heat map of clustered PSFs (Fig 3). The larger of the two clusters contains cells that are strongly up-regulated post-sffpike, which we call *bursty cells*.

*Analysis of post-spike filter parameters*: We analyzed six quantitative features of the PSFs that captured distinct aspects of temporal coding. We first reduced each PSF to its smooth component (see above). For a PSF *f*, we defined *rate-normalized power* as $P(f)=r\times \int_{0}^{t}f{\left(s\right)}^{2}ds$, where *r* is the mean firing rate of the cell and *t* is the duration of the PSF (~0.7 s). This normalization was chosen because the integral of the squared PSF (*raw power*) is strongly negatively correlated to the firing rate (data not shown). This is likely due to the fact there is a relatively constant amount of total firing rate modulation per cell, so low firing rate cells attribute more modulation *per spike* and thus have higher raw power.

To study ‘shape’ properties that are independent of power, we normalized each PSF to have total power equal to one:

$$f_{norm}=\frac{f}{\sqrt{\int_{0}^{t}f{\left(s\right)}^{2}ds}}.$$

We defined burstiness from the immediate post-spike interval value as $\int_{0}^{30ms}f_{norm}(s)ds$. This value is highly correlated with the instantaneous frequency (defined as 1/peak interspike interval) as well as percentage of <30ms ISIs (data not shown). For bursty cells (Fig 1), we defined the *theta peak* as the maximum value of *f*_*norm*_ in the at least 0.083s but not more than 0.167s (i.e. one theta cycle) post spike. We defined *theta peak time* as the time of the peak one theta cycle post spike. Likewise, the *theta trough* was the minimum value attained in the interval 0.042–0.083s, and *theta depth* was defined as theta peak minus theta trough. We defined *theta correlation* as the maximum linear correlation between *f*_*norm*_ and a theta-frequency cosine wave *g*_*A*,*ω*_(*t*) = *A*cos(*ωt*):

$$C_{\theta }=\underset{A,\omega }{\max}\left(corr\left(f_{norm},g_{A,\omega }\right)\right)$$

where the amplitude, *A*, is constrained to be positive and the frequency, *ω*, is constrained to be in the interval 6-12Hz. Lastly, we defined the *theta integral* as the integral of the normalized PSF between 83 ms and 250 ms, representing the area under the PSF for one theta cycle post spike. Predicted vs true autocorrelation shows that the models fit well across the whole population of neurons over a wide range of coherence values ranging from 5^th^ to 95^th^ percentile (Fig 3).

### Statistical analysis

We use generalized estimating equations (GEE) in SPSS (21.0 Chicago, III), which allows for within-animal correlations and the assumption of the most appropriate distribution for the data; all data distributions were visually assessed and the most appropriate link function was used. Goodness of fit was determined using the corrected quasi likelihood under independence model criterion and by the visual assessment of residuals. Two degrees of freedom was assumed based on a Wald chi-square within the GEE framework when comparing between three groups; in instances where two groups were compared (bursty vs refractory) there was one degree of freedom. We compared spatial coherence and PSF parameters across groups using GEE as multiple cells were obtained from individual animals. These cells are likely to be related to each other given that they are recorded in the same network. PSF shapes were compared between groups by down sampling the PSF to 10 ms bins across the entire timecourse of the PSF. Group by time interactions were compared to establish whether the PSFs all come from the same distribution. In addition to using *a priori* parameters of the PSF we also used a dimension reducing factor analysis in SPSS (21.0 Chicago, Ill) to identify factors in an unsupervised way. We obtained a Varimax rotated solution and generated factors for each cell for each solution. Factors with an eigenvalue of >1 were retained for further analysis. Cell counts were compared between groups using a negative binomial distribution appropriate for counts data. Histological data was also analyzed in this manner, with animal ID as the subject variable since sections from the same animal are not independent, neuron count per section as the dependent variable and group as a factor. All values reported are estimated marginal means +/- SEM. Multiple regression analyses within GEE with spatial coherence as the dependent variable and PSF parameters (or in a separate analysis, factors) and 2-way interaction terms as independent variables. Predicted values from the analyses were retained. Spearman and Pearson correlations were used to determine the relationships between observed and expected data and to obtain an objective measure of how much variance was explained by the regression analyses.

Effect sizes for all significant results were calculated using a standard Cohen’s d: mean 1-mean 2/pooled standard deviation.

## Supporting information

S1 Fig Exploratory principal component analysis of post-spike filters. (a) The coefficients of each time point for the first principal component (PC) of the post-spike filters (PSFs) show a high peak at t = 0 ms and smaller peaks at subsequent theta cycles (red dotted lines at t = 110 ms, 220 ms, 330 ms, and 440 ms). This demonstrates that whether a PSF conforms to this pattern or deviates from it is the dominant shape variation in the set of PSFs. This can be seen clearly in the heatmap of PSFs sorted by their score along the first PC (b). Note that strongly bursty, theta-modulated cells are at the bottom of the heatmap, while refractory, weakly theta-modulated cells are at the top. (c) The pairwise correlation matrix of the PSFs sorted as in panel b indicate two distinct clusters of PSFs with high in-group correlations and low/negative out-group correlations. The first and smaller cluster (top left) contains PSFs that are strongly anti-correlated with the second, larger cluster (bottom right). (d) The PSF scores of the two clusters identified by k-means clustering (k = 2; cf. Fig 4) differ significantly (t-test p = 3.6 x 10^−62^), with cluster 1 PSFs strongly anti-correlated with the first PC (panel a) and cluster 2 strongly correlated.
(TIF)

S2 Fig Exploratory correlation analysis of bursty post-spike filters and autocorrelation functions with spatial coherence. (a) The Spearman correlation coefficient between each PSF time point and spatial coherence has large peaks at t = 17 ms (C = 0.39) and t = 121 ms (C = 0.54), indicating that the immediate and one theta cycle post spike time periods contain information predictive of spatial coherence. (b) An analogous analysis of the smoothed autocorrelogram (ACG; smoothing window = 10 ms) shows high (C > 0.5) for all time points post spike. This demonstrates that the ACG predicts coherence using information varying on behavioral, not hippocampal circuit, timescales. In particular, the ACG retains information about spatial firing rate that must be corrected for when relating timing coding to spatial rate coding.
(TIF)

S3 Fig Autocorrelation of excluded cells. One rat was excluded from analysis because we could not confirm that its recorded cells were hippocampal in origin. A) An autocorrelogram of a representative example shows significant down-regulation immediately post-spike followed by overshooting and a return to baseline. Note further that there is no theta modulation. This pattern is characteristic of cortical neurons and is extremely distinct from the theta modulated, burst firing pattern we see in all other recordings in our experiment. Because our analysis centered on bursty cells, we removed this rat from subsequent analyses. B) Autocorrelograms of all cells from this animal were smoothed and scaled to make comparisons between cells (smoothed with a 20ms gaussian window and scaled by the area under the curve between 0s and 1s). The average scaled autocorrelogram recapitulates the characteristic shape in panel A. Error bars indicate standard error of the mean at each time point.
(TIF)

S4 Fig Table of variance explained by each of the 10 factor loadings. (TIF)

## Abbreviations

MCD: malformation of cortical development
EE: environmental enrichment
FST: fine spike timing
ACG: autocorrelogram
