# Supplementary figures and images for: Environmental enrichment normalizes hippocampal timing coding in a malformed hippocampus
Source: PLoS One. 2018 Feb 2;13(2):e0191488. doi: 10.1371/journal.pone.0191488 (PMC5796690; doi:10.1371/journal.pone.0191488)

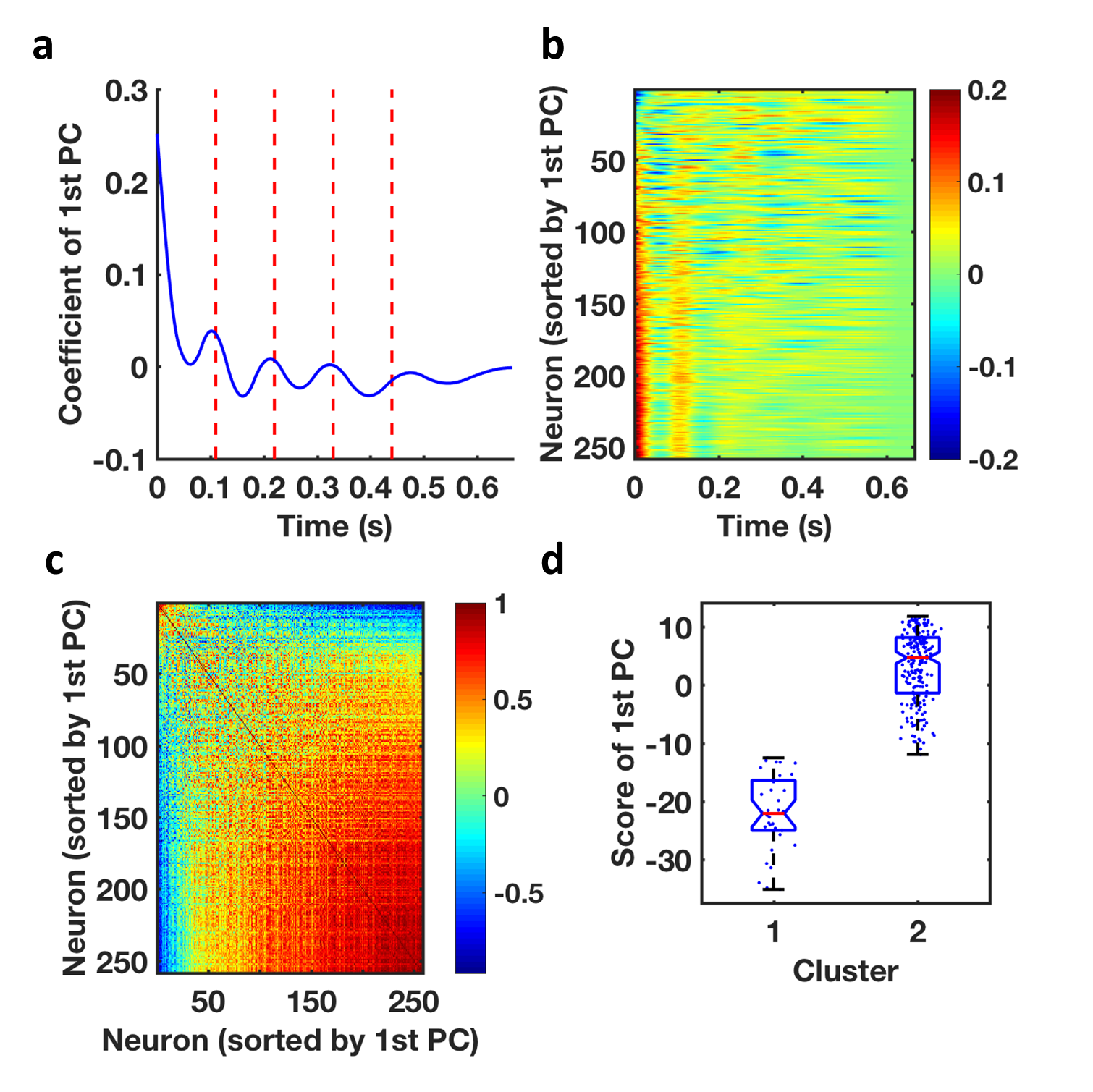

Supplement: S1 Fig — (a) The coefficients of each time point for the first principal component (PC) of the post-spike filters (PSFs) show a high peak at t = 0 ms and smaller peaks at subsequent theta cycles (red dotted lines at t = 110 ms, 220 ms, 330 ms, and 440 ms). This demonstrates that whether a PSF conforms to this pattern or deviates from it is the dominant shape variation in the set of PSFs. This can be seen clearly in the heatmap of PSFs sorted by their score along the first PC (b). Note that strongly bursty, theta-modulated cells are at the bottom of the heatmap, while refractory, weakly theta-modulated cells are at the top. (c) The pairwise correlation matrix of the PSFs sorted as in panel b indicate two distinct clusters of PSFs with high in-group correlations and low/negative out-group correlations. The first and smaller cluster (top left) contains PSFs that are strongly anti-correlated with the second, larger cluster (bottom right). (d) The PSF scores of the two clusters identified by k-means clustering (k = 2; cf. Fig 4) differ significantly (t-test p = 3.6 x 10^−62^), with cluster 1 PSFs strongly anti-correlated with the first PC (panel a) and cluster 2 strongly correlated. (TIF) [file pone.0191488.s001.tif]

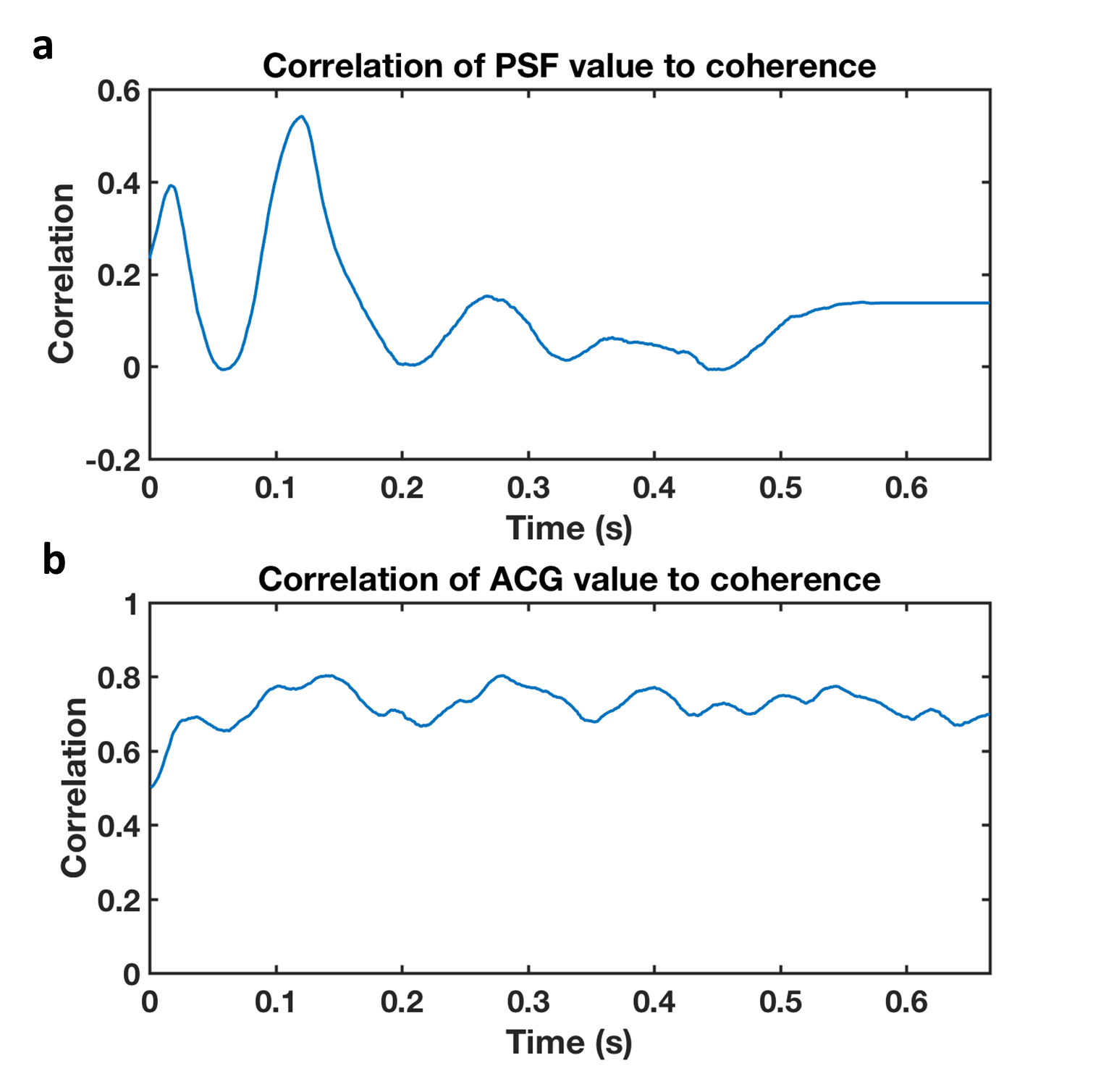

Supplement: S2 Fig — (a) The Spearman correlation coefficient between each PSF time point and spatial coherence has large peaks at t = 17 ms (C = 0.39) and t = 121 ms (C = 0.54), indicating that the immediate and one theta cycle post spike time periods contain information predictive of spatial coherence. (b) An analogous analysis of the smoothed autocorrelogram (ACG; smoothing window = 10 ms) shows high (C > 0.5) for all time points post spike. This demonstrates that the ACG predicts coherence using information varying on behavioral, not hippocampal circuit, timescales. In particular, the ACG retains information about spatial firing rate that must be corrected for when relating timing coding to spatial rate coding. (TIF) [file pone.0191488.s002.tif]

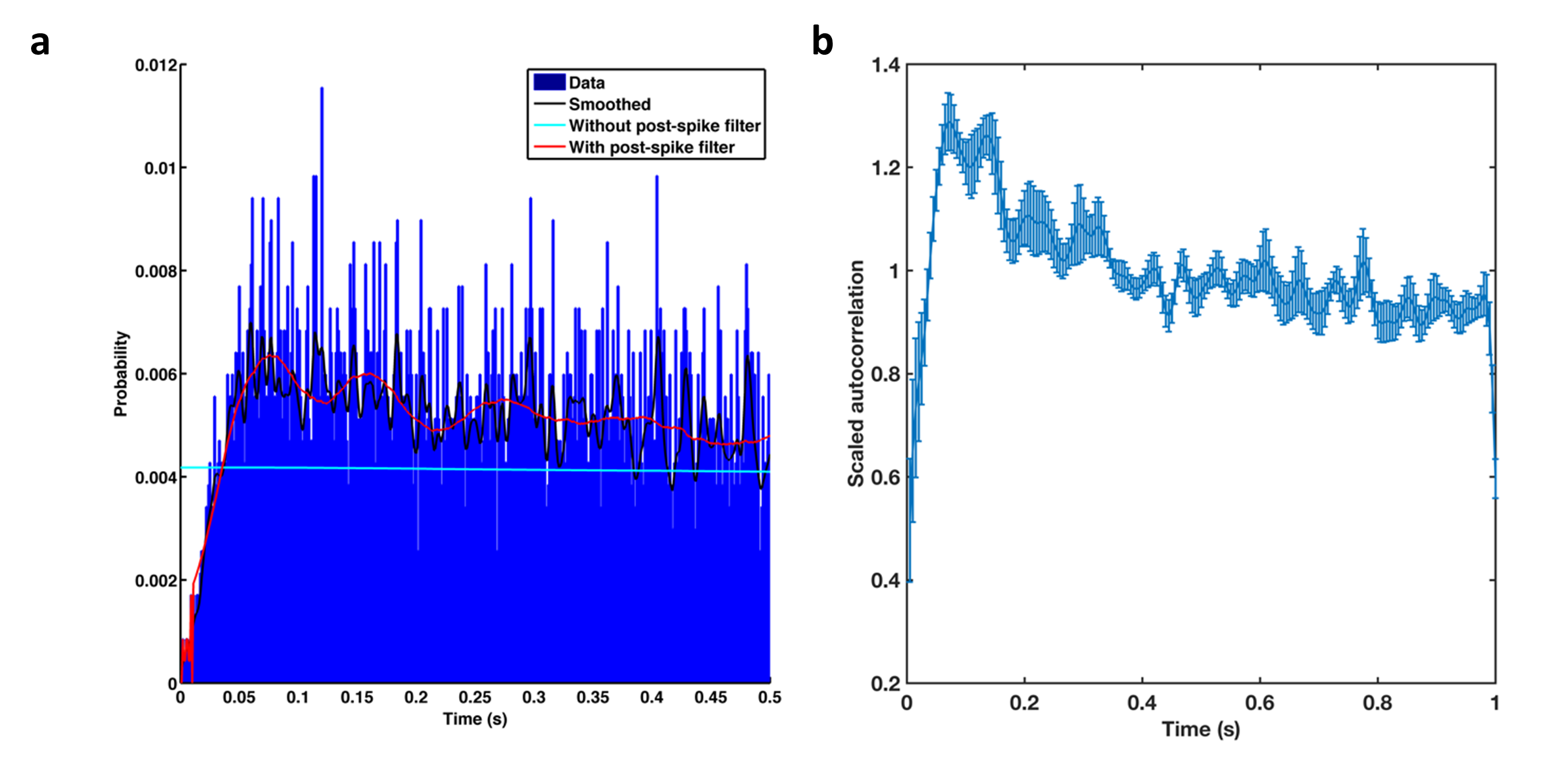

Supplement: S3 Fig — One rat was excluded from analysis because we could not confirm that its recorded cells were hippocampal in origin. A) An autocorrelogram of a representative example shows significant down-regulation immediately post-spike followed by overshooting and a return to baseline. Note further that there is no theta modulation. This pattern is characteristic of cortical neurons and is extremely distinct from the theta modulated, burst firing pattern we see in all other recordings in our experiment. Because our analysis centered on bursty cells, we removed this rat from subsequent analyses. B) Autocorrelograms of all cells from this animal were smoothed and scaled to make comparisons between cells (smoothed with a 20ms gaussian window and scaled by the area under the curve between 0s and 1s). The average scaled autocorrelogram recapitulates the characteristic shape in panel A. Error bars indicate standard error of the mean at each time point. (TIF) [file pone.0191488.s003.tif]

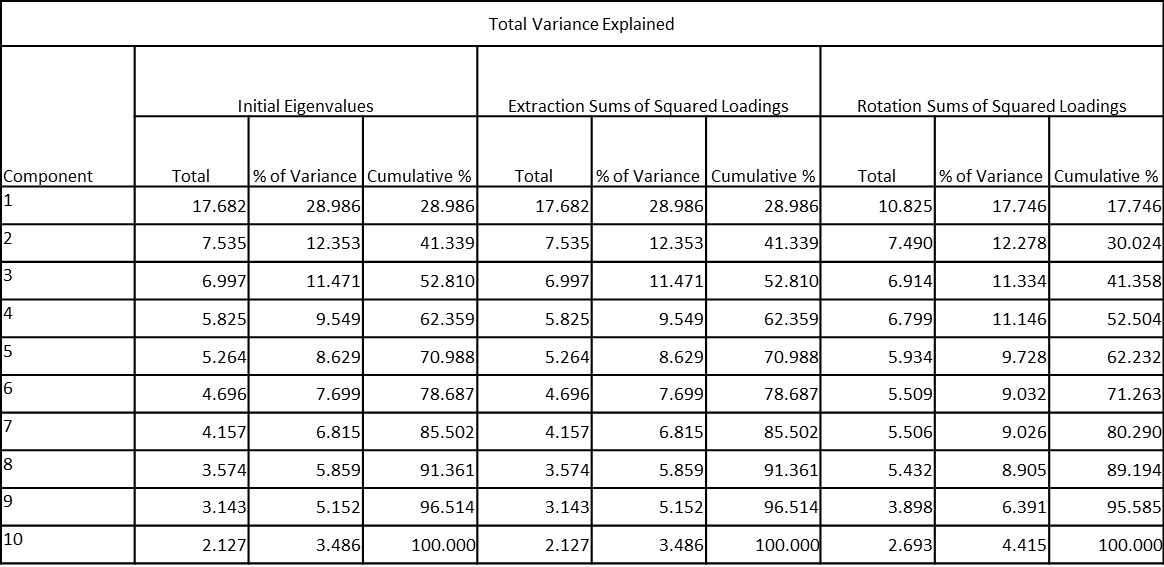

Supplement: S4 Fig — (TIF) [file pone.0191488.s004.tif]
